# Human macrophage response to the emerging enteric pathogen *Aeromonas veronii*: Inflammation, apoptosis, and downregulation of histones

**DOI:** 10.1080/21505594.2024.2440554

**Published:** 2024-12-11

**Authors:** Nicholas Naidovski, Sarah K. T. Chong, Fang Liu, Stephen M. Riordan, Michael C. Wehrhahn, Christopher Yuwono, Li Zhang

**Affiliations:** a School of Biotechnology and Biomolecular Sciences, University of New South Wales, Sydney, Australia; b Gastrointestinal and Liver Unit, Prince of Wales Hospital, University of New South Wales, Sydney, Australia; c Douglass Hanly Moir Pathology, a Sonic Healthcare Practice, Macquarie Park, NSW, Australia

**Keywords:** *Aeromonas*, *Aeromonas veronii*, *Escherichia coli*, macrophages, THP-1 macrophages, gastroenteritis

## Abstract

This study investigated the pathogenic mechanisms of *Aeromonas veronii* in macrophages. THP-1 derived macrophages were used as a human macrophage model and were treated with *A. veronii* strain AS1 isolated from intestinal biopsies of an IBD patient, or *Escherichia coli* strain K-12. RNA was extracted and subjected to RNA sequencing and comparative transcriptomic analyses. Protein levels of IL-8, IL-1β, IL-18, and TNFα were measured using ELISA, and apoptosis was assessed using caspase 3/7 assays. Both *A. veronii* AS1 and *E. coli* K-12 significantly upregulated the expression of many genes involving inflammation. At the protein level, *A. veronii* AS1 induced significantly higher levels of IL-8, TNFα, mature IL-18 and IL-1β than *E. coli* K-12, and led to greater elevation of caspase 3/7 activities. Both *A. veronii* AS1 and *E. coli* K-12 upregulated the expression of *CASP5*, but not other caspase genes. *A. veronii* AS1 significantly downregulated the expression of 20 genes encoding histone proteins that *E. coli* K-12 did not. The more profound pathogenic effects of *A. veronii* in inducing inflammation and apoptosis in macrophages than *E. coli* K-12 are consistent with its role as a human enteric pathogen. The upregulated expression of *CASP5* and increased release of IL-1β and IL-18 support the role of *CASP5* in activation of non-canonical inflammasome. The downregulation of histone genes by *A. veronii* suggests a unique impact on host cell gene expression, which may represent a novel virulence strategy. These findings advance the understanding of pathogenic mechanisms of the emerging human enteric pathogen *A. veronii.*

## Introduction

Members of the genus *Aeromonas* are gram-negative, rod-shaped, non-spore-forming, facultative anaerobes approximately 1–3 μm in size [1]. *Aeromonas* species are predominantly found in aquatic environments such as rivers, lakes, and streams, showing preference for lower saline waters [1,2]. These bacteria are both oxidase- and catalase-positive [3].

Several *Aeromonas* species have been implicated as the causative agents of various human diseases such as gastroenteritis, bacteraemia, wound infections, soft tissue infections, and respiratory infections, of which gastroenteritis is the most common [4–10]. *Aeromonas* species have recently been identified as the second most common bacterial enteric pathogen in Australia, after *Campylobacter* species [11]. *Aeromonas-*associated gastroenteritis usually presents as acute diarrhoea lasting less than two weeks. However, *Aeromonas* enteric infections also manifest as chronic diarrhoea lasting four or more weeks, dysentery, or colitis [1,10,12–14]. Compared to other enteric bacterial pathogens, *Aeromonas* caused enteric infections have a unique infection pattern, with three infection-peaks in young children, young adults, and individuals over 50 y old, respectively [11].

Patients with inflammatory bowel disease (IBD) are more susceptible to *Aeromonas* enteric infections [15]. IBD is a chronic inflammatory syndrome of the gastrointestinal tract, with Crohn’s disease and ulcerative colitis constituting its two major clinical forms [16]. IBD patients with *Aeromonas* infections are more likely to develop severe inflammation and the development of chronic distal colitis and ulcerative colitis proctitis in young adults following infection with *Aeromonas* has been recorded, suggesting that *Aeromonas* species may trigger the development of IBD [13,17–19].

Most of the human *Aeromonas* enteric infections are caused by four *Aeromonas* species, including *Aeromonas veronii, Aeromonas caviae, Aeromonas hydrophila*, and *Aeromonas dhakensis*. In some countries such as Pakistan and Bangladesh, *A. caviae* has been the most frequently isolated *Aeromonas* species from children with *Aeromonas* enteric infections [20]. In other countries such as Australia, the most commonly isolated *Aeromonas* species from patients with gastroenteritis was reported to be *A. veronii* [11].

The pathogenic mechanisms by which *Aeromonas* species cause human enteric diseases are largely unknown. In this study, we investigated the response in human macrophages to *A. veronii* through transcriptomic analysis, measurement of cytokine production, assessment of cell death and morphological changes. Macrophages play a key role in human innate immune responses to pathogens and causing inflammation [21]. The data from this study provide novel insights into understanding the human innate immune response against *A. veronii* infection and how this bacterium may mediate enteric disease in humans.

## Materials and methods

### Generation of THP-1 derived macrophages

The human leukaemic monocytic cell line THP-1 has been frequently used as a model for monocyte and macrophage functions and mechanisms [22]. In this study, we used THP-1 derived macrophages (THP-1 macrophages) as a human macrophage cell model to investigate the global gene response to *A. veronii.*

THP-1 cells (TIB-202; ATCC, Virginia, United States) were grown in RPMI 1640 medium, supplemented with 10% foetal bovine serum (FBS), 1 mM sodium pyruvate, 2.25 mg/L sodium bicarbonate, 10 mM *N*-2-hydroxyethylpiperazine-N-2-ethane sulphonic acid (HEPES) buffer, and 100 U/mL penicillin, and 100 μg/mL streptomycin in sterile T75 cm2 tissue culture flasks (Corning, New York, United States). This media was referred to as complete media. To allow the cells to proliferate, these flasks were then placed in a humidified incubator containing 5% CO_2_ and incubated at 37°C under aerobic conditions and maintained according to the procedures recommended by the ATCC. All reagents used were purchased from Thermo Fisher Scientific, CA, United States, except for FBS which was purchased from Sigma-Aldrich, NSW, Australia.

As previously described, for use in experiments THP-1 cells were cultured into welled tissue culture plates at a desired concentration in complete media containing 50 ng/mL phorbol 12-myristate 13-acetate (PMA) (Sigma-Aldrich, NSW, Australia) for 48 hours to allow differentiation into THP-1 macrophage cells. Once differentiated and adhered, the THP-1 macrophages were washed three times with Dulbecco’s phosphate-buffered saline (DPBS) and then incubated with fresh complete media for an additional 48 hours. Experiments were performed on the fifth day where THP-1 macrophages were washed three times with DBPS and complete media without penicillin streptomycin was added prior to bacterial treatment [23].

### Bacterial strains used in this study

This study used the *A. veronii* strain AS1, which was previously isolated from intestinal biopsies of a patient with IBD, and its completed genome sequence has been obtained [15]. *A. veronii* AS1 was cultured on horse blood agar (HBA) plates prepared using Blood Agar Base no. 2 (Thermo Fisher Scientific, CA, United States), supplemented with 6% (v/v) defibrinated horse blood. The inoculated plates were incubated for 24 hours at 37°C under aerobic conditions.

*Escherichia coli* strain K-12, a widely used commensal *E. coli* strain, was used as the bacterial control in this study. This strain was chosen as it has been extensively studied and characterized. It lacks many of the virulence factors found in pathogenic *E. coli* strains, making it an ideal bacterial control when studying host responses to pathogens [24]. Similarly, *E. coli* K-12 was cultured on HBA plates and incubated for 24 hours at 37°C under aerobic conditions.

### Comparative transcriptomic analysis of the gene expression responses to *A. veronii*

#### Pre-experimental work to determine the incubation time of THP-1 macrophages with *A. veronii* AS1 or *E. coli* K-12

To optimise experimental conditions for transcriptomic analysis, pre-experimental work was conducted. THP-1 macrophages (1 × 10^6^/well in 6-well culture plate) were treated with *A. veronii* AS1 or *E. coli* K-12 for 1, 2, 3, and 4 hours in triplicates at a multiplicity of infection (MOI) of 1. In triplicates, THP-1 macrophages without bacterial treatment were used as the negative control. At 4 hours it was noted that THP-1 macrophages treated with *A. veronii* AS1 were floating in the supernatant and RNA extraction yield was extremely low, indicative of cell death. At 2 hours, detached cells floating in the supernatant were minimal and this time point provided the best RNA quality. As such, the following conditions for subsequent transcriptomic experiments were used: THP-1 macrophages (1 × 10^6^/well in 6-well culture plate) were treated with *A. veronii* AS1 or *E. coli* K-12 for 2 hours in triplicates at a MOI of 1. THP-1 macrophages without bacterial treatment in triplicates were used as the negative control.

#### Total RNA extraction, library preparation, and RNA sequencing

Total RNA extraction was performed on THP-1 macrophages treated with *A. veronii* AS1 or *E. coli* K-12 and on control cells using the Isolate II RNA minikit (Bioline) following the manufacturer’s instructions. The purity and concentration of the RNA were determined using a NanoDrop spectrophotometer. The integrity of the RNA was evaluated using TapeStation, and the samples with an RNA integrity number of ≥7 were selected for subsequent analysis.

The RNA sequencing (RNA-seq) was performed by the Ramaciotti Centre for Genomics, University of New South Wales. In summary, libraries were created using the Illumina Stranded Total RNA Prep Ligation with Ribo-Zero Plus kit (Illumina) and then subjected to sequencing on the Illumina NovaSeq6000 platform.

#### Identification of differentially expressed genes

RNA-seq analysis was conducted using the publicly available nf-core RNA-seq pipeline v3.12.2 in Nextflow v23.10.0 [25–27]. Briefly, raw paired-end reads underwent quality assessment with FastQC v0.12.0 [28]. Following quality checks, the data were then processed with Trim Galore! v0.6.5 and subsequently aligned to the human reference genome GRCh38.p14 using STAR v2.7.11a [29,30]. Quantification of the aligned reads was performed using Salmon v1.10.2 [31]. The counts aligned per gene were utilised as input for the analysis of differential gene expression using the DESeq2 v1.40.2 R Bioconductor package. This was performed following default normalisation methods. We determined differential gene expression in THP-1-like macrophages treated with either *A. veronii* AS1 or *E. coli* K-12 by comparing it to non-treated cells, using the approach detailed in prior studies [32,33].

Biologically significant differentially expressed genes were identified using an adjusted *p* value (*p*-adj) threshold of <0.05 and a log_2_ fold change (log2FC) ≥1 or ≤−1. Statistical significance was assessed using Wald’s test and then adjusted using the Benjamini–Hochberg method embedded in the DESeq2 package. Visualisation of biologically significant differentially expressed genes was accomplished through a volcano plot and chromosome position representation using EnhancedVolcano package v1.18.0, ggmaplot v0.1.2 and chromoMap v4.1.1 in R [34–36].

#### Gene ontology enrichment and KEGG pathway mapper analysis

Biologically significant differentially expressed genes were analysed by performing gene ontology (GO) enrichment analysis through Enrichr. The use of Enrichr provides the user with information on how differentially expressed genes may influence cellular biological processes [37]. The top 10 enriched biological processes following the treatment of THP-1 macrophages with *A. veronii* AS1 or *E. coli* K-12 were identified and listed in descending order according to the *p* value calculated using the Fisher exact test [37]. Additionally, biologically significant up- and downregulated genes were parsed through the KEGG Mapper tool, to infer cellular functions and pathways from these genes [38].

#### Observation of THP-1 macrophage morphology

To evaluate the morphology of THP-1 macrophages at the conditions used for transcriptomic analysis, THP-1 cells were seeded at a density of 1 × 10^6^/well in 6-well culture plates and on coverslips, respectively, and differentiated into macrophages using PMA as described above. Cells were then treated with *A. veronii* AS1 or *E. coli* K-12 for 2 hours at an MOI of 1. Untreated THP-1 macrophages served as the negative control.

Following incubation, THP-1 macrophages cultured in 6-well culture plates were observed and imaged at 40**×** magnification using the EXOS XL Core Imaging System (Invitrogen). THP-1 macrophages cultured on coverslips were fixed with 3.6% paraformaldehyde, permeabilized with 0.1% Triton X-100, and blocked using 1% bovine serum albumin. F-actin was stained using Alexa Fluor 488 Phalloidin (Cell Signalling Technology), and nuclei were counterstained with Hoechst 33,342 (Thermo Fisher Scientific, CA, United States), both according to the manufacturer’s instructions. Stained cells were visualised using an Olympus BX61 fluorescence microscope at both 20**×** and 100**×** magnification.

### Examination of the production of interleukin 8 (IL-8), interleukin 1 beta (IL-1β), interleukin 18 (IL-18), and tumour necrosis factor alpha (TNFα) induced by *A. veronii* AS1 or *E. coli* K-12 in THP-1 macrophages

This study aimed to assess the pro-inflammatory potential of *A. veronii* AS1 in THP-1 macrophages using enzyme-linked immunosorbent assay (ELISA). THP-1 macrophages (1 × 10^6^/well in 6-well culture plates) were treated in triplicates with *A. veronii* AS1 or *E. coli* K-12 for 1, 2, and 3 hours at an MOI of 1. As triplicates, untreated THP-1 macrophages served as the negative control.

After the bacterial treatment period, supernatants from the THP-1 macrophage cultures were collected and then subjected to ELISA to measure the levels of IL-8, IL-1β, IL-18, and TNFα in triplicates, using commercially available ELISA kits (Invitrogen, CA, United States), following the manufacturer’s instructions.

### Quantification of *A. veronii* AS1 induced apoptosis of THP-1 macrophages

This study aimed to quantify the apoptotic potential of *A. veronii* AS1 in THP-1 macrophages. THP-1 macrophages (1 × 10^5^/well in 96-well black-walled culture plates) were treated in triplicates with *A. veronii* AS1 or *E. coli* K-12 for 1, 2, and 3 hours at an MOI of 1. As triplicates, untreated THP-1 macrophages served as the negative control. THP-1 macrophages were also treated with staurosporine (STS) in triplicates, serving as the positive control.

Caspase 3/7 activity, indicative of apoptosis, was measured using the CellEvent Caspase 3/7 Green ReadyProbes reagent (Invitrogen) according to the manufacturer’s instructions. Caspase 3/7 activity levels were measured in triplicates and recorded as fold changes relative to the negative control.

## Results

### Both *E. coli* K-12 and *A. veronii* AS1 altered the expression of a large number of genes in THP-1 macrophages

**Figure 1.**
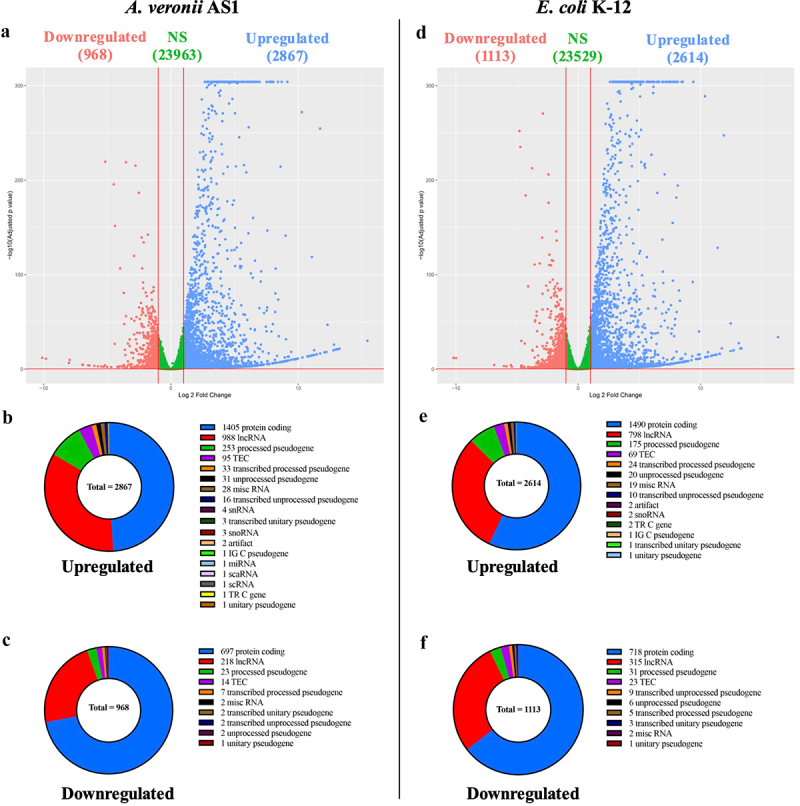
*A. veronii* AS1 and *E. coli* K12 upregulated and downregulated expression of genes in THP-1 macrophages.

*A. veronii* AS1 treatment induced a greater number of biologically significant upregulated transcripts than treatment with *E. coli* K-12 (2, 867 versus 2, 614), while treatment with *E. coli* K-12 resulted in a greater number of biologically significant downregulated transcripts than treatment with *A. veronii* AS1 (1, 113 versus 968) (Figure 1a,d). *A. veronii* AS1 and *E. coli* K-12 upregulated the expression of a large number of biologically significant protein coding genes (1, 405 and 1, 490, respectively) (Figure 1b,e). Similarly, *A. veronii* AS1 and *E. coli* K-12 upregulated a large number of biologically significant long non-coding RNAs (988 and 798, respectively) (Figure 1b,e). *A. veronii* AS1 downregulated 697 protein coding genes, while *E. coli* K-12 downregulated 718 biologically significant protein coding genes (Figure 1c,f). Both *A. veronii* AS1 and *E. coli* K-12 downregulated many biologically significant long non-coding RNAs (218 and 315, respectively) (Figure 1c,f).

(a and d) Volcano plots of the genes with altered expression in THP-1 macrophages when treated with *A. veronii* AS1 or *E. coli* K-12, respectively, for 2 hours as compared with the untreated control cells. Significant differentially expressed genes were identified using a *p*-adj cut-off of <0.05 and a log2FC of ≥1 or ≤−1. Infinite *p* values were assigned an arbitrarily small number (9.9E–305) to ensure inclusion within the plot. (b and e) Composition of biologically significant genes upregulated by *A. veronii* or *E. coli*, respectively. (c and f) Composition of biologically significant genes downregulated by *A. veronii* or *E. coli*, respectively. NS = not significant, LncRNA = long noncoding RNA, TEC = to be experimentally confirmed, misc RNA = miscellaneous RNA, snRNA = small nuclear RNA, snoRNA = small nucleolar RNA, miRNA = micro RNA, scaRNA = small Cajal body-specific RNA, scRNA = small conditional RNA.

### *A. veronii* AS1 and *E. coli* K-12 treatment of THP-1 macrophages resulted in the biologically significant upregulation of pro-inflammatory cytokine and chemokine encoding genes

Treatment of THP-1 macrophages with *A. veronii* AS1 or *E. coli* K-12 resulted in the biologically significant upregulation of 49 genes encoding pro-inflammatory cytokines and chemokines (Table 1). Of these genes, 44 were significantly upregulated by both bacterial species, and five were only significantly upregulated in THP-1 macrophages treated with *E. coli* K-12. Those only upregulated by *E. coli* K-12 were *TNFSF13B*, *TNFSF10, IL27*, *IL33*, and *CX3CL1*.

**Table 1.** Biologically significant upregulated cytokine and chemokine encoding genes following THP-1 macrophage treatment with *A. veronii* AS1 or *E. coli* K-12.

Gene Symbol	Gene Name	*A. veronii* AS1 log2FC (*p*-adj)	*E. coli* K-12 log2FC (*p*-adj)
*CCL1*	C-C motif chemokine ligand 1	3.47 (6.68E–04)	5.44 (1.33E–11)
*CCL15*	C-C motif chemokine ligand 15	2.08 (1.05E–03)	2.03 (7.30E–04)
*CCL19*	C-C motif chemokine ligand 19	9.10 (3.38E–09)	10.43 (1.57E–13)
*CCL2*	C-C motif chemokine ligand 2	5.01 (5.77E–127)	6.30 (1.35E–84)
*CCL20*	C-C motif chemokine ligand 20	7.53 (0)	7.20 (0)
*CCL22*	C-C motif chemokine ligand 22	3.15 (3.30E–170)	3.42 (2.69E–252)
*CCL3*	C-C motif chemokine ligand 3	6.42 (0)	6.22 (0)
*CCL3L3*	C-C motif chemokine ligand 3 like 3	9.16 (0)	8.56 (0)
*CCL4*	C-C motif chemokine ligand 4	10.29 (9.74E–273)	10.39 (1.37E–289)
*CCL4L2*	C-C motif chemokine ligand 4 like 2	11.72 (2.18E–255)	11.92 (4.60E–248)
*CCL5*	C-C motif chemokine ligand 5	2.96 (1.41E–266)	3.19 (0)
*CCL8*	C-C motif chemokine ligand 8	6.29 (2.71E–06)	9.93 (2.36E–16)
*CX3CL1^a^*	C-X3-C motif chemokine ligand 1	0.79 (1.99E–02)	3.57 (1.94E–02)
*CXCL1*	C-X-C motif chemokine ligand 1	8.71 (0)	8.45 (0)
*CXCL10*	C-X-C motif chemokine ligand 10	6.28 (5.75E–102)	9.38 (6.83E–93)
*CXCL11*	C-X-C motif chemokine ligand 11	4.60 (1.76E–28)	7.73 (1.78E–155)
*CXCL12*	C-X-C motif chemokine ligand 12	2.56 (3.69E–27)	3.08 (4.97E–49)
*CXCL13*	C-X-C motif chemokine ligand 13	3.92 (5.71E–08)	5.43 (6.88E–21)
*CXCL2*	C-X-C motif chemokine ligand 2	7.93 (0)	7.87 (0)
*CXCL3*	C-X-C motif chemokine ligand 3	7.87 (0)	7.43 (5.67E–06)
*CXCL6*	C-X-C motif chemokine ligand 6	5.72 (6.73E–30)	6.10 (2.54E–38)
*CXCL8*	C-X-C motif chemokine ligand 8	8.41 (0)	8.16 (0)
*CXCL9*	C-X-C motif chemokine ligand 9	4.40 (3.01E–08)	7.94 (7.84E–54)
*IFNL1*	Interferon lambda 1	7.21 (2.56E–05)	7.44 (5.67E–06)
*IFNβ1*	Interferon beta 1	12.22 (6.96E–19)	11.21 (8.08E–16)
*IL10*	Interleukin 10	3.99 (2.69E–57)	4.79 (2.96E–112)
*IL12β*	Interleukin 12 beta	6.99 (3.97E–22)	5.75 (7.40E–06)
*IL15*	Interleukin 15	4.03 (3.12E–50)	3.55 (4.53E–45)
*IL17C*	Interleukin 17 C	1.79 (1.13E–02)	1.79 (6.99E–03)
*IL18*	Interleukin 18	1.37 (9.94E–17)	1.21 (1.38E–14)
*IL1A*	Interleukin 1 alpha	8.21 (7.84E–81)	8.24 (6.07E–84)
*IL1β*	Interleukin 1 beta	7.85 (0)	7.71 (0)
*IL20*	Interleukin 20	6.49 (2.34E–04)	6.04 (1.21E–03)
*IL23A*	Interleukin 23 alpha	4.41(2.55E–126)	4.89 (1.03E–199)
*IL24*	Interleukin 24	1.91 (3.70E–04)	1.25 (2.14E–02)
*IL27*^*a*^	Interleukin 27	0.25 (4.20E–01)	3.77 (7.21E–04)
*IL32*	Interleukin 32	5.69 (1.28E–58)	6.13 (3.55E–78)
*IL33*^*a*^	Interleukin 33	0.91 (1.10E–01)	1.81 (3.69E–04)
*IL36G*	Interleukin 36 gamma	3.98 (2.25E–10)	4.95 (1.46E–21)
*IL6*	Interleukin 6	9.18 (6.95E–14)	10.62 (1.13E–18)
*IL7*	Interleukin 7	2.80 (2.39E–02)	3.93 (5.26E–03)
*LIF*	LIF interleukin 6 family cytokine	5.35 (4.22E–46)	5.25 (7.88E–46)
*TNF*	Tumour necrosis factor	7.52 (0)	7.52 (0)
*TNFSF10*^*a*^	TNF superfamily member 10	0.04 (8.97E–01)	1.70 (2.00E–35)
*TNFSF13B*^*a*^	TNF superfamily member 13b	0.62 (2.24E–02)	1.81 (2.93E–20)
*TNFSF15*	TNF superfamily member 15	4.07 (1.97E–271)	4.22 (9.71E–247)
*TNFSF18*	TNF superfamily member 18	2.62 (1.24E–25)	2.72 (2.48E–29)
*TNFSF8*	TNF superfamily member 8	7.13 (2.78E–05)	7.93 (5.71E–07)
*TNFSF9*	TNF superfamily member 9	2.42 (3.85E–74)	2.81 (2.97E–119)

^a^Genes only significantly upregulated in *E. coli* K-12 treated THP-1 macrophages.

Forty-nine biologically significantly upregulated cytokine and chemokine encoding genes (*p*-adj <0.05, log2FC ≥ 1) were identified through RNA-seq analysis of THP-1 macrophages treated with *A. veronii* AS1 or *E. coli* K-12 for 2 hours. Forty-four of these genes are significantly upregulated by both bacteria, with five only significantly upregulated by *E. coli* K-12. These are *TNFSF13B*, *TNFSF10, IL27*, *IL33*, and *CX3CL1*. Gene symbols and names were recorded. The log2FC and *p*-adj for both bacterial species are recorded and are listed adjacent. Statistical significance was assessed using Wald’s test and then adjusted using the Benjamini–Hochberg method.

### *A. veronii* AS1 and *E. coli* K-12 treatment of THP-1 macrophages resulted in the biologically significant upregulation of genes involved in the NOD-like receptor signalling pathway

Treatment of THP-1 macrophages with *A. veronii* AS1 or *E. coli* K-12 resulted in the biologically significant upregulation of 53 genes involved in the NOD-like receptor (NLR) signalling pathway (Table 2). Of these genes, 8 were only significantly upregulated in *E. coli* K-12 treated cells and were *AIM2, CASR, GBP1, GBP7, MEFV, OAS3, RIPK1*, and *STAT2. GBP7* was not identified in *A. veronii* AS1 treated cells. One gene, *PYDC5*, was only significantly upregulated in *A. veronii* AS1 treated cells.

**Table 2.** Regulation of genes involved in the NOD-like receptor signalling pathway upon treatment of THP-1 macrophages with *A. veronii* AS1 or *E. coli* K-12.

Gene Symbol	Gene Name	*A. veronii* AS1 log2FC (*p-*adj)	*E. coli* K-12 log2FC (*p-*adj)
*AIM2*^*b*^	Absent in melanoma 2	0.55 (4.89E–02)	1.11 (5.28E–08)
*BCL2L1*	BCL2 like 1	1.21 (4.72E–42)	1.49 (1.41E–60)
*BIRC2*	Baculoviral IAP repeat containing 2	2.10 (1.84E–165)	2.17 (2.43E–167)
*BIRC3*	Baculoviral IAP repeat containing 3	7.74 (0)	7.90 (0)
*CASP5*	Caspase 5	2.02 (1.35E–15)	2.32 (2.83E–26)
*CASR*^*b*^	Calcium sensing receptor	0.51 (1.18E–01)	1.10 (4.11E–02)
*CCL2*	C-C motif chemokine ligand 2	5.01 (5.77E–127)	6.30 (1.35E–84)
*CCL5*	C-C motif chemokine ligand 5	2.96 (1.41E–266)	3.19 (0)
*CXCL1*	C-X-C motif chemokine ligand 1	8.71 (0)	8.45 (0)
*CXCL2*	C-X-C motif chemokine ligand 2	7.93 (0)	7.87 (0)
*CXCL3*	C-X-C motif chemokine ligand 3	7.87 (0)	7.43 (0)
*CXCL8*	C-X-C motif chemokine ligand 8	8.41 (0)	8.16 (0)
*GABARAPL1*	GABA type A receptor associated protein like 1	1.56 (7.73E–56)	1.39 (3.19E–52)
*GBP1*^*b*^	Guanylate binding protein 1	0.69 (9.43E–08)	1.81 (8.69E–69)
*GBP2*	Guanylate binding protein 2	4.83 (0)	4.70 (0)
*GBP3*	Guanylate binding protein 3	1.18 (4.16E–23)	1.51 (2.31E–44)
*GBP4*	Guanylate binding protein 4	1.98 (8.20E–83)	2.78 (1.39E–215)
*GBP5*	Guanylate binding protein 5	3.89 (1.62E–295)	4.43 (0)
*GBP7*^*b*^	Guanylate binding protein 7		2.31 (1.80E–02)
*IFNAR2*	Interferon alpha and beta receptor subunit 2	1.31 (3.94E–77)	1.09 (4.83E–54)
*IFNB1*	Interferon beta 1	12.22 (6.96E–19)	11.21 (8.08E–16)
*IL18*	Interleukin 18	1.37 (9.94E–17)	1.21 (1.38E–14)
*IL1B*	Interleukin 1 beta	7.85 (0)	7.71 (0)
*IL6*	Interleukin 6	9.18 (6.95E–14)	10.62 (1.13E–18)
*IRF7*	Interferon regulatory factor 7	1.73 (1.64E–19)	3.28 (3.37E–79)
*IRF9*	Interferon regulatory factor 9	1.91 (5.05E–114)	2.65 (1.26E–257)
*JUN*	Jun proto-oncogene, AP-1 transcription factor subunit	1.07 (3.20E–27)	1.21 (3.36E–36)
*MAP1LC3A*	Microtubule associated protein 1 light chain 3 alpha	1.16 (2.18E–07)	1.32 (2.40E–12)
*MAPK10*	Mitogen-activated protein kinase 10	2.75 (1.21E–13)	2.57 (1.01E–13)
*MEFV*^*b*^	MEFV innate immunity regulator, pyrin	0.73 (2.34E–08)	1.14 (7.96E–22)
*NAMPT*	Nicotinamide phosphoribosyl transferase	3.93 (0)	4.02 (0)
*NFKB1*	Nuclear factor kappa beta subunit 1	3.92 (0)	4.09 (0)
*NFKBIA*	NFKB inhibitor alpha	4.85 (0)	4.92 (0)
*NFKBIB*	NFKB inhibitor beta	1.50 (1.21E–28)	1.60 (4.36E–34)
*NLRP3*	NLR family pyrin domain containing 3	1.36 (5.00E–74)	1.25 (9.51E–64)
*NOD2*	Nucleotide binding oligomerization domain containing 2	1.72 (3.78E–24)	2.17 (2.32E–49)
*OAS1*	2”−5”-oligoadenylate synthetase 1	1.81 (3.38E–61)	2.85 (0)
*OAS2*	2”−5”-oligoadenylate synthetase 2	2.17 (1.98E–72)	3.40 (0)
*OAS3*^*b*^	2”−5”-oligoadenylate synthetase 3	0.79 (3.15E–13)	2.17 (8.11E–213)
*P2RX7*	Purinergic receptor P2X 7	2.99 (5.11E–245)	2.62 (4.49E–191)
*PANX1*	Pannexin 1	2.27 (1.12E–173)	2.47 (1.22E–216)
*PYDC5^a^*	Pyrin domain containing 5	1.24 (1.17E–02)	0.62 (2.01E–01)
*RIPK1*^*b*^	Receptor interacting serine/threonine kinase 1	0.99 (5.26E–24)	1.09 (4.70E–27)
*RIPK2*	Receptor interacting serine/threonine kinase 2	3.76 (2.24E–293)	3.89 (0)
*STAT1*	Signal transducer and activator of transcription 1	1.11 (1.02E–27)	2.42 (5.98E–229)
*STAT2*^*b*^	Signal transducer and activator of transcription 2	0.67 (7.43E–15)	1.85 (1.27E–157)
***TAB 2***	TGF-beta activated kinase 1 (MAP3K7) binding protein 2	1.32 (3.81E–28)	1.27 (1.82E–27)
*TANK*	TRAF family member associated NFKB activator	2.32 (5.40E–174)	2.07 (8.68E–128)
*TICAM1*	TIR domain containing adaptor molecule 1	1.91 (5.41E–29)	2.20 (1.26E–37)
*TNF*	Tumour necrosis factor	7.52 (0)	7.52 (0)
*TNFAIP3*	TNF alpha induced protein 3	5.53 (0)	5.59 (0)
*TRAF3*	TNF receptor associated factor 3	2.19 (3.44E–204)	2.14 (4.45E–202)
*TYK2*	Tyrosine kinase 2	1.02 (8.61E–16)	1.17 (1.72E–21)

^a^Genes only significantly upregulated in *A. veronii* AS1 treated THP-1 macrophages.

^b^Genes only significantly upregulated in *E. coli* K-12 treated THP-1 macrophages.

Fifty-three biologically significantly upregulated genes (*p*-adj <0.05, log2FC ≥ 1) involved in the NOD-like receptor signalling pathway were identified through RNA-seq analysis of THP-1 macrophages treated with *A. veronii* AS1 or *E. coli* K-12 for 2 hours. Eight of these genes were uniquely significantly upregulated in *E. coli* K-12 treated cells and were *AIM2, CASR, GBP1, GBP7, MEFV, OAS3, RIPK1*, and *STAT2* while one gene, *PYDC5*, was uniquely upregulated in *A. veronii* AS1 treated cells. *GBP7* was not identified in *A. veronii* AS1 treated cells. Gene symbols and names were recorded. The log2FC and *p*-adj for both bacterial species are recorded and are listed adjacent. Statistical significance was assessed using Wald’s test and then adjusted using the Benjamini–Hochberg method.

### *A. veronii* AS1 induced clustered histone downregulation on chromosome 6 not seen in *E. coli* K-12 treated THP-1 macrophages

**Figure 2.**
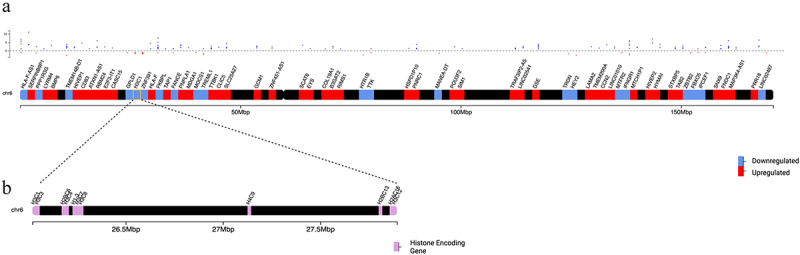
*A. veronii* AS1 induced the downregulation of histone encoding genes on chromosome 6.

Transcriptomic analysis of THP-1 macrophages treated with *A. veronii* AS1 revealed the biologically significant differential expression of 2, 102 protein coding genes. Among these, 188 (9%) were located on chromosome 6 (Figure 2a). Of these 188 genes, 51 (27%) genes were biologically significantly downregulated, with 22 (43%) encoding clustered histones. Only two of these histone encoding genes on chromosome 6, *H3C8* and *H2BC10*, were biologically significantly downregulated in THP-1 macrophages treated with *E. coli* K-12 (Table 3). All biologically significant downregulated histone encoding genes identified in this study were located on chromosome 6.

(a) All (188) biologically significant differentially expressed genes (*p*-adj <0.05, log2FC ≥1 or ≤−1) located on chromosome 6 following treatment of THP-1 macrophages with *A. veronii* AS1 for 2 hours, with the first gene of each locus labelled. (b) All downregulated histone encoding genes identified in THP-1 macrophages treated with *A. veronii* AS1. THP-1 macrophages treated with *A. veronii* AS1 showed the biologically significant downregulation 22 histone encoding genes, accounting for 43% of all downregulated genes located on chromosome 6. All biologically significant downregulated histone encoding genes identified in this study were located on chromosome 6. Scatter plot represents the log2FC of all 188 biologically significant differentially expressed genes located on chromosome 6.

**Table 3.** Significantly downregulated histone encoding genes following treatment of THP-1 macrophages with *A. veronii* AS1 or *E. coli* K-12.

Gene Symbol	Gene Name	*A. veronii* AS1 log2FC (*p-*adj)	*E. coli* K-12 log2FC (*p*-adj)
*H1–3*	H1.3 linker histone, cluster member	−1.12 (3.58E–28)	−0.70 (3.27E–13)
*H2AC13*	H2A clustered histone 13	−1.03 (4.64E–10)	−0.67 (2.00E–04)
*H2AC16*	H2A clustered histone 16	−1.15 (2.44E–08)	−0.80 (6.17E–05)
*H2AC17*	H2A clustered histone 17	−1.03 (7.85E–17)	−0.56 (3.55E–06)
*H2AC4*	H2A clustered histone 4	−1.43 (6.95E–14)	−0.43 (1.93E–01)
*H2AC7*	H2A clustered histone 7	−1.07 (9.34E–08)	−0.67 (8.84E–04)
*H2BC10^a^*	H2B clustered histone 10	−1.63 (1.01E–28)	−1.37 (9.29E–19)
*H2BC13*	H2B clustered histone 13	−1.01 (5.83E–10)	−0.64 (4.72E–05)
*H2BC14*	H2B clustered histone 14	−1.11 (2.60E–12)	−0.72 (3.04E–08)
*H2BC17*	H2B clustered histone 17	−1.12 (3.76E–23)	−0.54 (1.95E–06)
*H2BC6*	H2B clustered histone 6	−1.03 (8.10E–18)	−0.81 (3.65E–10)
*H2BC9*	H2B clustered histone 9	−1.15 (2.68E–17)	−0.97 (8.53E–12)
*H3C1*	H3 clustered histone 1	−1.09 (4.24E–09)	−0.97 (4.86E–07)
*H3C11*	H3 clustered histone 11	−1.04 (8.48E–07)	−0.70 (1.28E–04)
*H3C12*	H3 clustered histone 12	−1.42 (1.02E–16)	−0.99 (1.85E–13)
*H3C2*	H3 clustered histone 2	−1.03 (5.71E–18)	−0.73 (2.32E–12)
*H3C3*	H3 clustered histone 3	−1.21 (5.10E–35)	−0.91 (1.63E–25)
*H3C4*	H3 clustered histone 4	−1.03 (2.72E–12)	−0.79 (3.32E–10)
*H3C7*	H3 clustered histone 7	−1.03 (1.44E–4)	−0.34 (4.75E–01)
*H3C8*^*a*^	H3 clustered histone 8	−1.70 (1.49E–18)	−1.15 (2.32E–15)
*H4C2*	H4 clustered histone 2	−1.04 (9.50E–08)	−0.35 (1.70E–01)
*H4C9*	H4 clustered histone 9	−1.60 (2.36E–19)	−0.87 (2.73E–06)

^a^Genes significantly downregulated by both *A. veronii* AS1 or *E. coli* K-12 treated THP-1 macrophages.

Twenty-two biologically significantly downregulated histone encoding genes (*p*-adj <0.05, log2FC ≤ −1) were identified through RNA-seq analysis of THP-1 macrophages treated with *A. veronii* AS1 or *E. coli* K-12 for 2 hours. Two of these genes (*H3C8* and *H2BC10*) are significantly downregulated by both bacteria, with 20 only significantly downregulated in *A. veronii* AS1 treated cells. Gene symbols and names were recorded. The log2FC and *p*-adj for both bacterial species are recorded and are listed adjacent. Statistical significance was assessed using Wald’s test and then adjusted using the Benjamini–Hochberg method.

### The histone genes significantly downregulated by *A. veronii* AS1 were associated with the neutrophil extracellular trap pathway

Pathway analysis identified 23 significantly downregulated genes by *A. veronii* AS1 that were associated with the neutrophil extracellular trap (NET) pathway. These 23 genes included the 22 histone genes identified above, and an additional gene *SIGLEC9* which encodes sialic acid binding Ig like lectin 9 (Table S1).

### Both *A. veronii* AS1 and *E. coli* K-12 induced the biologically significant upregulation of *CASP5* in THP-1 macrophages

Transcriptomic data of THP-1 macrophages following treatment with *A. veronii* AS1 or *E. coli* K-12 were analysed. We identified 10 caspase encoding genes, of which only *CASP5* was biologically significantly differentially expressed, and was shared by both bacteria (Table 4).

**Table 4.** Treatment of THP-1 macrophages with *A. veronii* AS1 or *E. coli* K-12 results in the biologically significant upregulation of *CASP5.*

Gene Symbol	Gene Name	*A. veronii* AS1 log2FC (*p*-adj)	*E. coli* K-12 log2FC (*p*-adj)
*CASP1*	Caspase 1	0.03 (8.72E–01)	0.41 (6.69E–04)
*CASP2*	Caspase 2	−0.74 (4.21E–13)	−0.80 (3.15E–17)
*CASP3*	Caspase 3	0.61 (1.04E–09)	0.61 (4.06E–13)
*CASP4*	Caspase 4	0.36 (8.27E–04)	0.55 (9.10E–08)
*CASP5*^*a*^	Caspase 5	2.02 (1.35E–15)	2.32 (2.83E–26)
*CASP6*	Caspase 6	−0.95 (7.97E–04)	−0.76 (3.63E–03)
*CASP7*	Caspase 7	0.37 (6.81E–03)	0.76 (1.51E–10)
*CASP8*	Caspase 8	0.24 (7.51E–02)	−0.16 (2.68E–01)
*CASP9*	Caspase 9	−0.30 (9.38E–02)	−0.11 (6.07E–01)
*CASP10*	Caspase 10	0.50 (1.61E–05)	0.90 (1.77E–16)

^a^Genes significantly upregulated by both *A. veronii* AS1 or *E. coli* K-12 treated THP-1 macrophages.

Ten genes encoding for caspases were identified through RNA-seq analysis of THP-1 macrophages treated with *A. veronii* AS1 or *E. coli* K-12 for 2 hours. Of these 10, only *CASP5* was biologically significantly upregulated (*p*-adj <0.05, log2FC ≥ 1). Gene symbols and names were recorded. The log2FC and *p*-adj for both bacterial species are recorded and are listed adjacent. Statistical significance was assessed using Wald’s test and then adjusted using the Benjamini–Hochberg method.

### *A. veronii* AS1 downregulated cytoskeletal reorganization, regulation of lipoprotein particle clearance and protein localization to the chromosome

**Figure 3.**
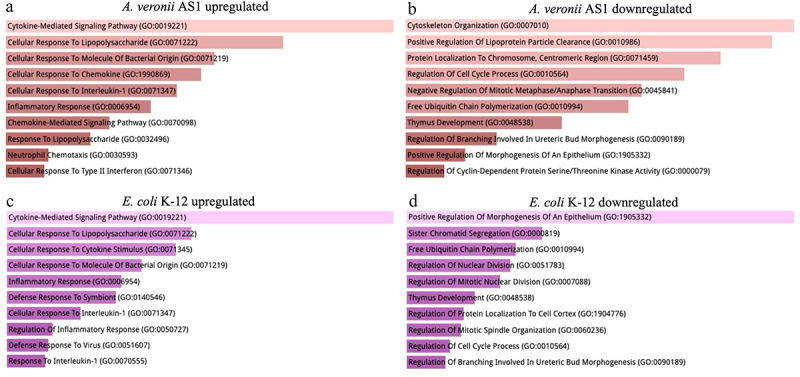
The impact of *A. veronii* AS1 or *E. coli* K-12 on biological processes in THP-1 macrophages.

The top 10 significantly enriched GO biological process terms ranked by *p* value in descending order are displayed in Figure 3. Both *A. veronii* AS1 and *E. coli* K-12 upregulated biological processes mainly concerned with inflammation and the inflammatory response such as the upregulation of cytokine-mediated signalling pathways. Dissimilarly, *A. veronii* AS1 treatment uniquely downregulated cytoskeleton organization, positive regulation of lipoprotein particle clearance, and protein localization to the centromeric region of the chromosome while *E. coli* K-12 treatment downregulated the positive regulation of morphogenesis of an epithelium as well as other dissimilar processes.

Using all biologically significant differentially expressed genes as input (*p*-adj <0.05, log2FC of ≥1 or ≤ −1), the top 10 Gene Ontology terms enriched in the biological processes category are provided, arranged in descending order based on their *p* values, calculated using the Fisher exact test. Treatment with *A. veronii* AS1 or *E. coli* K-12 of THP-1 macrophages led to the upregulation of biological processes primarily associated with inflammation and the inflammatory response, notably cytokine-mediated signalling pathways. *A. veronii* AS1 downregulated biological processes involved in cytoskeleton organization, positive regulation of lipoprotein particle clearance and protein localization to the centromeric region of the chromosome. In contrast, *E. coli* K-12 treatment strongly downregulated the positive regulation of the morphogenesis of an epithelium.

### Impact of *A. veronii* AS1 or *E. coli* K-12 treatment on the morphology and viability of THP-1 macrophages

**Figure 4.**
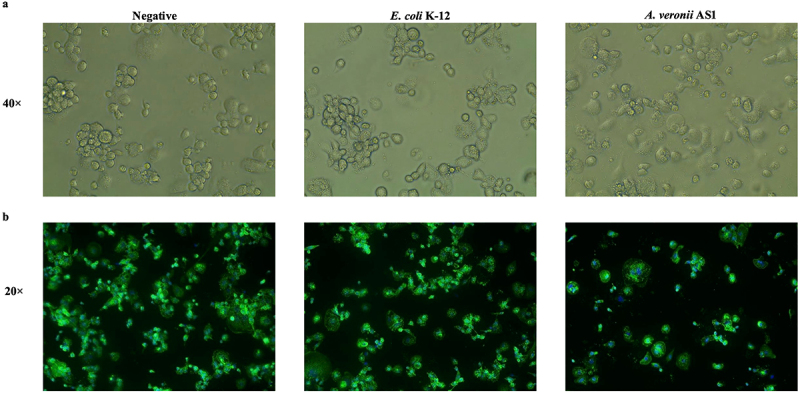
Morphology and viability of THP-1 macrophages following treatment with *A. veronii* AS1 or *E. coli* K-12.

After incubation with *A. veronii* AS1 or *E. coli* K-12 treatment for 2 hours, THP-1 macrophage cells cultured in 6-well culture plates remained adherent, and their morphology and density were similar to that of the untreated negative control cells (Figure 4a). However, for THP-1 macrophages cultured on coverslips and subjected to fluorescence stains, the density of cells treated with *A. veronii* AS1 decreased compared to those treated with *E. coli* K-12 and the negative control cells (Figure 4b). Among the adherent THP-1 macrophages on the coverslips, the morphology of cells treated with *A. veronii* AS1 was comparable to those treated with *E. coli* K-12 and the negative control (Fig S1).

To evaluate the morphology of THP-1 macrophages at the condition used for transcriptomic analysis, THP-1 macrophages were treated with *A. veronii* AS1 or *E. coli* K-12 for 2 hours at multiplicity of infection 1. Untreated THP-1 macrophages served as the negative control. (a) THP-1 macrophage cells cultured in 6-well culture plates remained adherent, and their morphology and density were similar to that of the untreated negative control cells. (b) THP-1 macrophages cultured on coverslips, treated with *A. veronii* AS1 or *E. coli* K-12 and subjected to the fluorescence stains Alexa Fluor 488 Phalloidin and Hoechst 33,342 showed decreased cell density when compared to those treated with *E. coli* K-12 and the negative control cells.

### Evaluating the production of IL-8, IL-1β, IL-18, and TNFα induced by *A. veronii* AS1 or *E. coli* K-12 in THP-1 macrophages

#### Evaluating IL-8 and TNFα production in THP-1 macrophages following treatment with *A. veronii* AS1 or *E. coli* K-12

**Figure 5.**
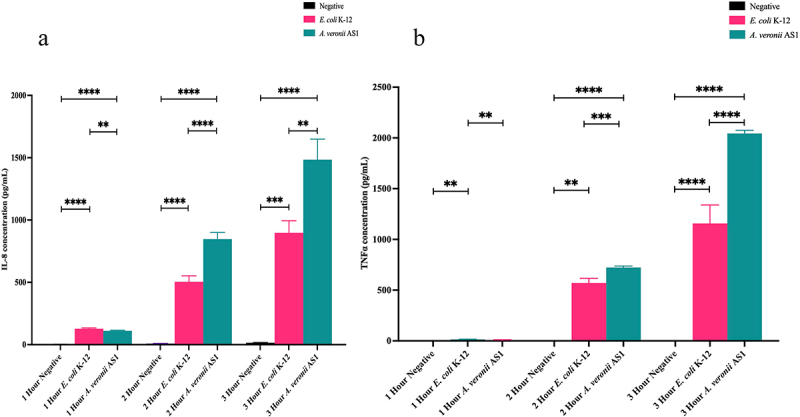
Production of IL-8 and TNFα in THP-1 macrophages induced by *A. veronii* AS1 or *E. coli* K-12 treatment.

Cell culture supernatants collected following treatment with *A. veronii* AS1 or *E. coli* K-12 after 1, 2, and 3 hours of treatment at MOI 1 were subjected to ELISA to determine IL-8 and TNFα production. After 1, 2, and 3 hours both *A. veronii* AS1 and *E. coli* K-12 induced detectable changes in IL-8 production. The levels of IL-8 produced by *A. veronii* AS1 at 1, 2, and 3 hours were 111.50 ± 4.07, 847.66 ± 53.11, and 1484.73 ± 163.3 pg/mL, respectively (Figure 5a). The levels of IL-8 produced by *E. coli* K-12 at 1, 2, and 3 hours were 128.27 ± 6.09, 504.51 ± 48, and 897.04 ± 96.63 pg/mL, respectively (Figure 5b).

Concentrations of IL-8 (a) and TNFα (b) in THP-1 macrophages were measured by sandwich ELISA following 1, 2, and 3 hours of treatment with *A. veronii* AS1 or *E. coli* K-12 at multiplicity of infection 1. A detectable level of IL-8 production following *A. veronii* AS1 treatment was observed at 1 (111.50 ± 4.07 pg/mL), 2 (847.66 ± 53.11 pg/mL), and 3 hours (1484.73 ± 164.3 pg/mL) post treatment. A detectable level of IL- 8 production following *E. coli* K-12 treatment was also detected at 1 (128.27 ± 6.09 pg/mL), 2 (504.51 ± 48.00 pg/mL), and 3 hours (897.04 ± 96.63 pg/mL) post treatment. A detectable level of TNFα production following *A. veronii* AS1 treatment was observed at 1 (3.93 ± 6.81 pg/mL), 2 (723.95 ± 13.06 pg/mL), and 3 hours (2044.27.63 ± 30.87 pg/mL) post treatment. A detectable level of TNFα production following *E. coli* K-12 treatment was also detected at 1 (15.34 ± 1.63 pg/mL), 2 (570.41 ± 45.57 pg/mL), and 3 hours (1156.87 ± 182.0 pg/mL) post treatment. The final values are presented as the average of triplicate samples with standard deviation (SD). *p* values are represented by asterisks (*). ** = *p* value < 0.01, *** = *p* value < 0.001, **** = *p* value < 0.0001. Statistical analysis was performed by one-way analysis of variance with Dunnett’s test.

After 1, 2, and 3 hours, both *A. veronii* AS1 and *E. coli* K-12 induced detectable changes in TNFα production. The levels of TNFα produced by *A. veronii* AS1 at 1, 2, and 3 hours were 3.93 ± 6.81, 723.95 ± 13.06, and 2044.27.63 ± 30.87 pg/mL, respectively (Figure 5b). The levels of TNFα produced by *E. coli* K-12 at 1, 2, and 3 hours were 15.34 ± 1.63, 570.41 ± 45.57, and 1156.87 ± 182.0 pg/mL, respectively (Figure 5b).

#### Evaluating IL-1β and IL-18 production in THP-1 macrophages following treatment with *A. veronii* AS1 or *E. coli* K-12

**Figure 6.**
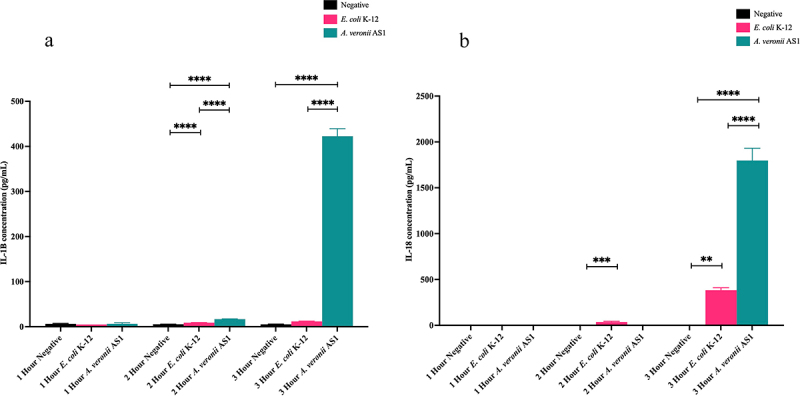
Production of IL-1β and IL-18 in THP-1 macrophages induced by *A. veronii* AS1 or *E. coli* K-12 treatment.

Cell culture supernatants collected following treatment with *A. veronii* AS1 or *E. coli* K-12 after 1, 2, and 3 hours of treatment at MOI 1 were subjected to ELISA to determine IL-1β and IL-18 production. After 1, 2, and 3 hours both *A. veronii* AS1 and *E. coli* K-12 induced detectable changes in IL-1β. The levels of IL-1β produced by *A. veronii* AS1 at 1, 2, and 3 hours were 6.41 ± 2.51, 16.82 ± 0.54, and 422.63 ± 16.7 pg/mL, respectively (Figure 6a). The levels of IL-1β produced by *E. coli* K-12 at 1, 2, and 3 hours were 4.85 ± 0.06, 8.89 ± 0.38, and 11.98 ± 0.41 pg/mL, respectively (Figure 6a).

Concentrations of IL-1β (a) and IL-18 (b) in THP-1 macrophages were measured by sandwich ELISA following 1, 2, and 3 hours of treatment with *A. veronii* AS1 or *E. coli* K-12 at multiplicity of infection 1. (a) Detectable levels of IL-1β production following *A. veronii* AS1 treatment were observed at 1 (6.41 ± 2.51 pg/mL), 2 (16.82 ± 0.54 pg/mL), and 3 hours (422.63 ± 16.7 pg/mL) post treatment. Detectable levels of IL-1β production following *E. coli* K-12 treatment were also detected at 1 (4.85 ± 0.06 pg/mL), 2 (8.89 ± 0.38 pg/mL), and 3 hours (11.98 ± 0.41 pg/mL) post treatment. (b) Detectable levels of IL-18 production following *A. veronii* AS1 treatment were observed at 3 hours (1797.31 ± 134.3) post treatment. Detectable levels of IL-18 production following *E. coli* K-12 treatment were observed at 2 hours (36.35 ± 8.4) and 3 hours (383.9 ± 25.8) post treatment. The final values are presented as the average of triplicate samples with standard deviation (SD). *p* values are represented by asterisks (*). ** = *p* value < 0.01, *** = *p* value < 0.001, **** = *p* value < 0.0001. Statistical analysis was performed by one-way analysis of variance with Dunnett’s test.

After 3 hours, *A. veronii* AS1 induced detectable changes in IL-18 production. The levels of IL-18 produced by *A. veronii* AS1 at 3 hours was 1797.31 ± 134.3 pg/mL (Figure 6b). After 2 and 3 hours, *E. coli* K-12 induced detectable changes in IL-18 production. The levels of IL-18 produced by *E. coli* K-12 at 2 and 3 hours were 36.35 ± 8.4 and 383.9 ± 25.8 pg/mL, respectively (Figure 6b).

### Quantification of the caspase 3/7 activity of THP-1 macrophages following treatment with *A. veronii* AS1 or *E. coli* K-12

**Figure 7.**
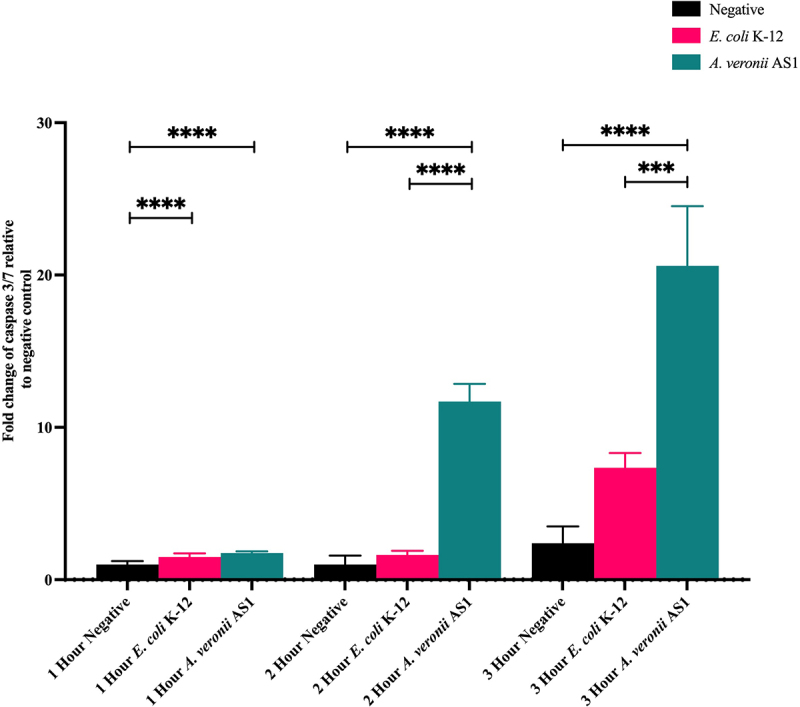
Caspase 3/7 activity induced by *A. veronii* AS1 or *E. coli* K-12 treatment of THP-1 macrophages.

The ability of *A. veronii* AS1 or *E. coli* K-12 to cause apoptosis in THP-1 macrophages was quantified. Caspase 3/7 activity was measured after THP-1 macrophages were treated with *A. veronii* AS1 or *E. coli* K-12 at an MOI of 1 for 1, 2, and 3 hours, and the fold change relative to the negative control was recorded. At 1, 2, and 3 hours post infection, *E. coli* K-12 induced caspase 3/7 levels of 1.49 ± 0.24, 1.62 ± 0.28 and 7.34 ± 0.98, respectively (Figure 7). At 1, 2, and 3 hours post infection, *A. veronii* AS1 induced caspase 3/7 levels of 1.75 ± 0.11, 11.69 ± 1.17, and 20.60 ± 3.92, respectively (Figure 7). The fold changes in caspase 3/7 activity in STS-treated THP-1 macrophages at 1, 2, and 3 hours were 3.67 ± 0.60, 6.66 ± 0.60, and 0.92 ± 3.24, respectively.

Caspase 3/7 activity in THP-1 macrophages was quantified by a caspase 3/7 assay following 1, 2, and 3 hours of treatment with *A. veronii* AS1 or *E. coli* K-12 at multiplicity of infection 1. At 1 hour, caspase 3/7 activity was observed in *E. coli* K-12 (1.49 ± 0.25) and *A*. *veronii* AS1 (1.75 ± 0.11) treated cells. By 2 hours, caspase 3/7 activity increased in *E. coli* K-12 (1.62 ± 0.28) and *A. veronii* AS1 (11.69 ± 1.17) treated cells. At 3 hours, caspase 3/7 activity further increased in *E. coli* K-12 (7.34 ± 0.98) and *A. veronii* AS1 (20.60 ± 3.92) treated cells. The fold changes in caspase 3/7 activity in STS-treated THP-1 macrophages at 1, 2, and 3 hours were 3.67 ± 0.60, 6.66 ± 0.60, and 0.92 ± 3.24, respectively. The data are presented as the average of triplicate samples with SD. Final values are expressed as the fold change when compared to the negative control. *p* values are represented by asterisks (*). ** = *p* value < 0.01, *** = *p* value < 0.001, **** = *p* value < 0.0001. Statistical analysis was performed by one-way analysis of variance with Dunnett’s test.

## Discussion

In this study, using THP-1 macrophages as a model of human macrophages, we investigated the global gene responses to the emerging human enteric pathogen *A. veronii* strain AS1 through comparative transcriptomic analyses, using *E. coli* strain K-12 as a control bacterium. We also measured the protein levels of proinflammatory cytokines IL-8, IL-1β, IL-18, and TNFα produced by THP-1 macrophages induced by *A. veronii* AS1 and *E. coli* K-12. Furthermore, we examined the apoptosis in THP-1 macrophages induced by *A. veronii* AS1 and *E. coli* K-12.

Both *A. veronii* AS1 and *E. coli* K-12 altered the expression of a large number of genes (Figure 1). The biological processes impacted by the biologically significant upregulated genes by *A. veronii* AS1 and *E. coli* K-12 were similar, primarily involved in inflammatory responses such as cytokine-mediated signalling pathways, cellular response to lipopolysaccharide, and cellular response to molecules of bacterial origin, consistent with the functions of macrophages (Figure 3). Further examination of upregulated genes encoding cytokines, chemokines and intracellular microbial detection receptors showed that the relevant genes biologically significantly upregulated by *A. veronii* AS1 were largely also biologically significantly upregulated by *E. coli* K-12, suggesting that common bacterial components have contributed to the upregulation of these inflammation and microbial sensor genes (Tables 1 and 2).

Despite the similarities in upregulation of the inflammation genes at the mRNA level, *A. veronii* AS1 induced a significantly higher level of IL-8, IL-1β, IL-18, and TNFα proteins released into the cell culture supernatants than *E. coli* K-12 (Figure 5 and 6). Production of IL-1β by THP-1 derived macrophages in response to *A. veronii* was also reported previously by other researchers [39]. IL-1β and IL-18 are key indicators of inflammasome activation, with mature IL-1β and IL-18 released after being cleaved by caspase 1, an enzyme activated by the inflammasome complex. Both IL-1β and IL-18 are proinflammatory cytokines [40,41]. While IL-1β is broadly involved in various inflammatory processes, IL-18 is more involved in promoting Th1 responses and enhancing IFNγ production [42,43]. Further, both IL-8 and TNFα need to be cleaved in order for their active soluble form to be secreted [44–46]. Both *A. veronii* AS1 and *E. coli* K-12 upregulated the expression of these genes, however, *A. veronii* AS1 induced significantly higher level of the mature forms of these cytokines at protein levels, suggesting that *A. veronii* may possess virulence factors that can contribute to the maturation of these proinflammatory cytokines.

The expression of the *CASP5* gene, which encodes caspase 5, was significantly upregulated by both *A. veronii* AS1 and *E. coli* K-12 (Table 4). Caspase 5 is an inflammatory caspase that has been found to mediate the one-step non-canonical inflammasome activation, mediating IL-1β and IL-18 release [47]. Identified in this study, caspase 5 may have contributed to the maturation of IL-1β and IL-18 in macrophage response to *A. veronii* AS1 and *E. coli* K-12.

*A. veronii* AS1 significantly downregulated 22 protein-coding genes encoding for clustered histones in THP-1 macrophages, while *E. coli* K-12 only significantly downregulated two of these genes (Table 3). These histone encoding genes are all located on chromosome 6 which contains genes essential for immunity and inflammation, including the major histocompatibility complex crucial for antigen presentation to T cells (Figure 2) [48]. The histones H2A, H2B, H3, and H4 play essential roles in packaging DNA into chromatin and regulating cell replication [49]. Normally, specific subsets of macrophages are capable of self-renewal, enabling them to continue performing their tissue-supportive functions like maintaining tissue homoeostasis and pathogen clearance [50,51]. The significant downregulation of these histones suggests that *A. veronii* AS1 may be capable of damaging the host immune response via the impairment of macrophage self-renewal through the downregulation of core histones. The significant downregulation of histone encoding genes has also been observed in intestinal epithelial cells infected with *A. veronii* AS1 but not in those infected with *E. coli* K-12, suggesting that this may be a pathogenic mechanism unique to *A. veronii* [15].

Further, this study identified 23 significantly downregulated genes in *A. veronii* AS1 treated THP-1 macrophages that were associated with the NET formation pathway, 22 of which were histone encoding genes (Table S1). NETs are characterised as large, extracellular web-like structures composed of histones, DNA-fibres and anti-microbial proteins that can capture, neutralize and kill bacteria, and thus form a component of the innate immune response [52–54]. More recently, it has been found that macrophages are able to produce their own variation of extracellular traps, termed macrophage extracellular traps (METs) [55]. The production of these METs has been shown to be stimulated by *E. coli* and the pathogenic bacterium *Staphylococcus aureus* in which they showed microbicidal activity [56]. The significant downregulation of these histone encoding genes associated with the NET formation pathway suggests that *A. veronii* may possess mechanisms to evade or suppress this component of the host innate immune response, particularly as histones form a core part of NETs and METs [55]. Moreover, as only three genes associated with the NET pathway were identified in *E. coli* K-12 cells (Table S1), this process may be unique to *A. veronii* infection.

*A. veronii* AS1 induced a significantly higher level of apoptosis in THP-1 macrophages than *E. coli* K-12, as indicated by the levels of caspase 3/7 (Figure 7). Previous studies examining the effects of *A. veronii* AS1 on intestinal epithelial cells also demonstrated elevated caspase 3/7 activity, suggesting that bacterium-induced apoptosis of host cells may contribute to *A. veronii* pathogenicity [15]. Bacterial-induced apoptosis of host immune cells is recognised as a key virulence mechanism of bacterial pathogens [57]. Despite the increased caspase 3/7 activity in THP-1 macrophages, *A. veronii* AS1 did not cause obvious morphological changes in cells cultured in 6-well cell culture plates after 2-hours of treatment, suggesting that these cells were at the early stage of apoptosis (Figure 4A). However, under the same experimental conditions, fewer THP-1 macrophages treated with *A. veronii* AS1 remained adherent to coverslips compared to those treated with *E. coli* K-12 and the negative control cells (Figure 4B). Due to the different surface properties of cell culture plates and coverslips, damaged THP-1 macrophages are more likely to detach from coverslips. The cells that remain adherent to the coverslips displayed similar morphologies, indicating they remain healthy (Figure S1).

**Figure 8.**
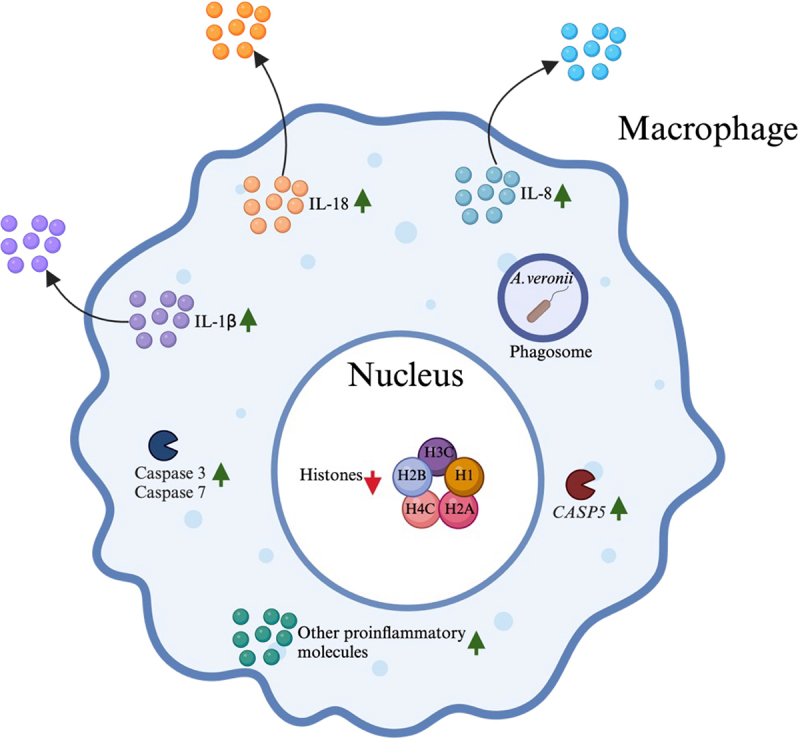
Summary of the pathogenic effects and mechanisms induced by *A. veronii* bacterial treatment of human macrophages.

In summary, our study shows that *A. veronii* AS1 elicits distinct responses in THP-1 macrophages, characterised by the release of higher levels of pro-inflammatory cytokines, histone gene downregulation, and increased apoptosis, compared to commensal *E. coli*. The main findings of this study are summarised in Figure 8, which contribute to the understanding of pathogenic mechanisms of the emerging human enteric pathogen *A. veronii*.

The *A. veronii* strain AS1 induces various detrimental effects in human macrophages. These effects include the upregulation of multiple proinflammatory molecules such as IL-8 and TNFα, as well as IL-1β and IL-18, the downregulation of expression of histone encoding genes, the upregulation of *CASP5*, and the induction of macrophage apoptosis through the intrinsic pathway through increasing caspase 3/7 activities.

## Supplementary Material

Fig S1.jpg

Table S1.docx

## Data Availability

The data discussed in this publication have been deposited in NCBI’s Gene Expression Omnibus and are accessible through GEO Series accession number GSE273835 (https://www.ncbi.nlm.nih.gov/geo/query/acc.cgi?acc=GSE273835) [58].
