# Supplementary figures and images for: Human macrophage response to the emerging enteric pathogen Aeromonas veronii: Inflammation, apoptosis, and downregulation of histones
Source: Virulence. 2024 Dec 11;16(1):2440554. doi: 10.1080/21505594.2024.2440554 (PMC11702953; doi:10.1080/21505594.2024.2440554)

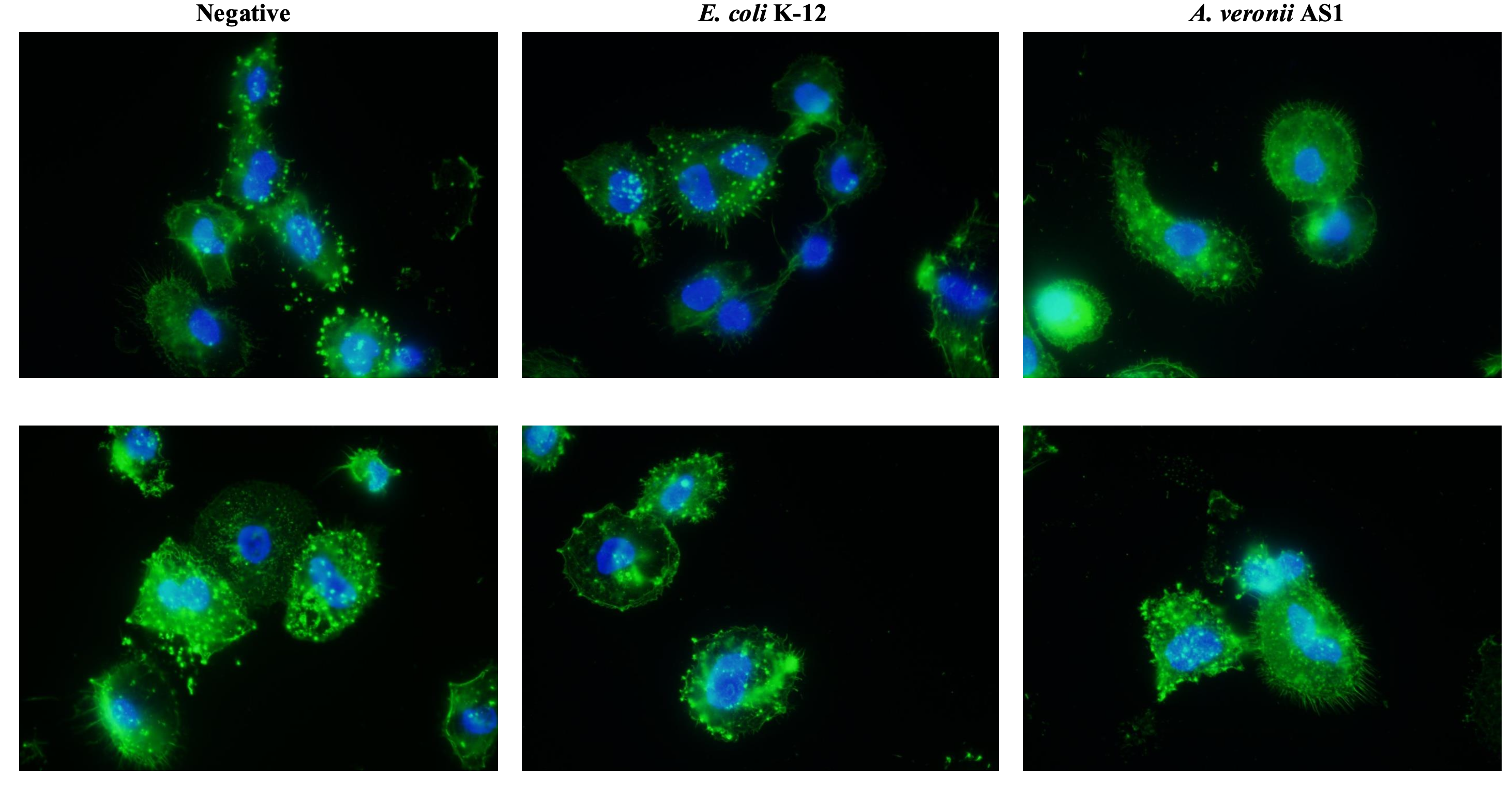

Supplement: Fig S1.jpg [file KVIR_A_2440554_SM2176.jpg]
