# Supplementary material for: Human macrophage response to the emerging enteric pathogen Aeromonas veronii: Inflammation, apoptosis, and downregulation of histones
Source: Virulence. 2024 Dec 11;16(1):2440554. doi: 10.1080/21505594.2024.2440554 (PMC11702953; doi:10.1080/21505594.2024.2440554)
Supplement: Table S1.docx [file KVIR_A_2440554_SM2175.docx]

**Table S1. Regulation of genes involved in the neutrophil extracellular trap pathway upon treatment of THP-1 macrophages with *A. veronii* AS1 or *E. coli* K-12**

| **Gene Symbol** | **Gene Name** | ***A. veronii* AS1 log2FC (*p-*adj)** | ***E. coli* K-12 log2FC (*p*-adj)** |
| --- | --- | --- | --- |
| *H3C8* | H3 clustered histone 8 | -1.70 (1.49E-18) | -1.15 (2.32E-15) |
| *H2BC10* | H2B clustered histone 10 | -1.63 (1.01E-28) | -1.37 (9.29E-19) |
| *SIGLEC9* | Sialic acid binding Ig like lectin 9 | -1.60 (3.69E-13) | -1.77 (9.10E-16) |
| *H4C9* | H4 clustered histone 9 | -1.60 (2.36E-19) |  |
| *FCGR3A* | Fc gamma receptor IIIa | -1.48 (1.0E-2) |  |
| *H2AC4* | H2A clustered histone 4 | -1.43 (6.95E-14) |  |
| *H3C12* | H3 clustered histone 12 | -1.42 (1.02E-16) |  |
| *H3C3* | H3 clustered histone 3 | -1.21 (5.10E-35) |  |
| *H2AC16* | H2A clustered histone 16 | -1.15 (2.44E-08) |  |
| *H2BC9* | H2B clustered histone 9 | -1.15 (2.68E-17) |  |
| *H1-3* | H1.3 linker histone, cluster member | -1.12 (3.58E-28) |  |
| *H2BC17* | H2B clustered histone 17 | -1.12 (3.76E-23) |  |
| *H2BC14* | H2B clustered histone 14 | -1.11 (2.60E-12) |  |
| *H3C1* | H3 clustered histone 1 | -1.09 (4.24E-09) |  |
| *H2AC7* | H2A clustered histone 7 | -1.07 (9.34E-08) |  |
| *H3C11* | H3 clustered histone 11 | -1.04 (8.48E-07) |  |
| *H4C2* | H4 clustered histone 2 | -1.04 (9.50E-08) |  |
| *H2AC13* | H2A clustered histone 13 | -1.03 (4.64E-10) |  |
| *H2AC17* | H2A clustered histone 17 | -1.03 (7.85E-17) |  |
| *H2BC6* | H2B clustered histone 6 | -1.03 (8.10E-18) |  |
| *H3C2* | H3 clustered histone 2 | -1.03 (5.71E-18) |  |
| *H3C4* | H3 clustered histone 4 | -1.03 (2.72E-12) |  |
| *H3C7* | H3 clustered histone 7 | -1.03 (1.44E-4) |  |
| *H2BC13* | H2B clustered histone 13 | -1.01 (5.83E-10) |  |

Twenty-three significant downregulated genes (*p*-adj < 0.05, log2FC ≤ -1) associated with the neutrophil extracellular trap (NET) pathway were identified through RNA-seq analysis of THP-1 macrophages treated with *A. veronii* AS1 for 2 hours. After 2 hours of treatment, three significantly downregulated genes associated with the NET pathway were identified in *E. coli* K-12 treated cells. Gene symbols and names were recorded. The log2FC and *p*-adj for both bacterial species are recorded and are listed adjacent. Statistical significance was assessed using Wald’s test and then adjusted using the Benjamini-Hochberg method.
